# Anxiety in Cancer Patients during ^18^F-FDG PET/CT Low Dose: A Comparison of Anxiety Levels before and after Imaging Studies

**DOI:** 10.1155/2017/3057495

**Published:** 2017-03-14

**Authors:** Ana Grilo, Lina Vieira, Elisabete Carolino, Cátia Oliveira, Carolina Pacheco, Maria Castro, Juan Alonso

**Affiliations:** ^1^Department of Humanities and Social Sciences, Escola Superior de Tecnologia da Saúde de Lisboa, Instituto Politécnico de Lisboa, Lisboa, Portugal; ^2^Centro de Investigação em Ciências Psicológica, Faculdade de Psicologia, Universidade de Lisboa, Lisboa, Portugal; ^3^Área Cientifica de Medicina Nuclear, Escola Superior de Tecnologia da Saúde de Lisboa, Instituto Politécnico de Lisboa, Lisboa, Portugal; ^4^Instituto de Biofísica e Engenharia Biomédica, Faculdade de Ciências da Universidade de Lisboa, Lisboa, Portugal; ^5^Department of Natural Sciences, Escola Superior de Tecnologia da Saúde de Lisboa, Instituto Politécnico de Lisboa, Lisboa, Portugal; ^6^Escola Superior de Tecnologia da Saúde de Lisboa, Instituto Politécnico de Lisboa, Lisboa, Portugal; ^7^Servicio de Medicina Nuclear, Instituto Tecnológico de Servicios Sanitarios, Madrid, Spain; ^8^Servicio de Medicina Nuclear, Hospital General Universitario “Gregorio Marañon”, Madrid, Spain; ^9^Sociedad Española de Medicina Nuclear e Imagen Molecular, Madrid, Spain

## Abstract

*Objective*. Assessing the level of anxiety in oncology patients who underwent ^18^F-FDG PET/CT low dose scan and identifying the main reasons that generate anxiety.* Material and Method*. The study included 81 cancer patients submitted to the ^18^F-FDG PET/CT low dose scan. Patients filled in the Scan Experience Questionnaire and the State-Trait Anxiety Inventory (STAI) before and after ^18^F-FDG PET/CT low dose scan.* Results*. Substantial levels of anxiety were detected both before and after ^18^F-FDG PET/CT low dose scan (STAI mean > 30), with a significant increase in the state of anxiety after scan performance (*p* < 0.0001, Median_pre_ = 31.1, and Median_pos_ = 33.0). ^18^F-FDG PET/CT low dose results are the main cause of anxiety both before (79.1%) and after (86.9%) the scan. The information provided by staff both before and on the ^18^F-FDG PET/CT low dose day was classified mostly as completely understandable (70.5% and 75.3%, resp.) and as very useful (70.5% and 72.6%, resp.) and correlated positively with patients' overall satisfaction with NM Department (*r*_*S*_ = 0.372, *p* = 0.004 and *r*_*S*_ = 0.528,* p* = 0.000, resp.), but not with anxiety levels.* Conclusions*. Patients perceive high levels of anxiety during the ^18^F-FDG PET/CT low dose scan and the concern with scan results was pointed out as the main factor for that emotional reaction.

## 1. Introduction

Positron Emission Tomography/Computed Tomography (PET/CT) low dose with ^18^F-fluor-2-deoxi-D-glucose (^18^F-FDG) and other radiopharmaceuticals (e.g., ^11^C-colina, ^11^C-acetato, and ^11^C-metionina) is of increasing interest in the study of the cancer patient since it is indicated in differential diagnosis, follow-up, and prognostic and therapeutic plan related to cancer diseases [1, 2].

Anxiety can be defined as a complex reaction to situations when perceived by patient as dangerous even if just under an uncertain circumstance. It can take many forms, as psychic, physiological, and behavioral components [3]. This emotional reaction is often felt by cancer patients during ^18^F-FDG PET/CT low dose scans. There are many factors that can generate anxiety during ^18^F-FDG PET/CT low dose procedures, as the use of radiopharmaceuticals, which produce radiation and because of it many patients can experience anxiety [4]. The equipment is a source of great anxiety in claustrophobic patients. Anxiety is also related to what the scan represents to the patient not only because of the procedure itself but also due to the results of the scanning and what it can represent to him or her, especially in a situation of oncology disease diagnostic or recrudescence of a cancer previously diagnosed [5]. Patients with hearing problems and difficulty in speaking may experience higher levels of anxiety because of the limited communication between them and health professionals [5, 6] (i.e., nurses, technologists of nuclear medicine, and physicians). High levels of anxiety can result in a poor image quality due to patient movement during procedure, which increases the uptake in the brown adipose tissue and in the muscle, leading to image false-positives [5, 7–9]. For that reason, the patient must rest after the injection of the radiopharmaceutical allowing the muscles to relax [9].

^18^F-FDG PET/CT low dose scan is prolonged and involves transmission of lots of information to the patient about radiopharmaceutical, scan procedure, between 30- and 60-minute waiting times. Therefore, effective communication is important because it allows the seizure and understanding of such information [5]. Some studies show that informed consent, including brief information about the risk factors and potential adverse reactions of the exam, reduces the level of anxiety, whereas detailed information before the procedure increases the anxiety level [10]. This fact is independent of the sociocultural level of the individual since a highly literate patient may also have difficulty in interpreting medical information [10].

The provision of adequate information should take the patient's needs into account, thus contributing to the increase of compliance, the reduction of symptoms, such as anxiety and fear, and the increase of customer satisfaction [11, 12]. The feeling of dissatisfaction means that the patient does not verbalize his or her concerns and his or her vulnerability and feels less relaxed. The assessment of patient satisfaction is difficult to quantify or even to define [13]. So all along health professionals should remind them of relevant information and clarify any doubts to the patient to make sure he or she is still, does not move, and is safe during the following examination, in anamnesis [14].

However research pointed out a few causes of anxiety; little evidence is currently available regarding the impact of information and the satisfaction with Nuclear Medicine Department on the levels of anxiety of patients when going through ^18^F-FDG PET/CT low dose scan. By providing detailed understanding of the levels of anxiety during ^18^F-FDG PET/CT low dose scan, educational efforts can be focused towards reducing anxiety related to ^18^F-FDG PET/CT low dose which could potentially interfere with patients' satisfaction and diagnostic accuracy. Therefore, this study aims to evaluate the level of anxiety in cancer patients who underwent PET/CT low dose scan with ^18^F-FDG and identify the main reasons that generate anxiety among these patients.

## 2. Material and Methods

### 2.1. Design

A cross-sectional prospective study was performed in two separate Nuclear Medicine (NM) Departments in the Iberian Peninsula, between 1st of April and 31st of May 2015, in cancer patients of different types (lung, breast, prostatic, and lymphoma) with clinical indication to do the ^18^F-FDG PET/CT low dose scan.

### 2.2. Participants and Settings

First authorization was requested from the boards of the two NM Departments. All eligible patients were previously notified with the purpose of the study, the protection of their personal information, the voluntary nature of participation, and the possibility to withdraw from the study at any time. Data collection commenced once the patient's written informed consent was obtained.

The convenience sample consisted of 95 patients who were scheduled for ^18^F-FDG PET/CT low dose. All patients complied with the inclusion following criteria: (1) over 18 years and (2) clinical indication for oncology reasons (lung, breast, prostatic, and lymphoma) to conduct the studies of ^18^F-FDG PET/CT low dose. Exclusion criteria included (1) patients with significant communication disabilities that would affect their ability to respond to questionnaires, (2) patients with a history of psychiatric illness, (3) patients in poor condition who were unable to cooperate, and (4) patients who scored more than 45 in State-Trait Anxiety Inventory survey (STAI-S) (see below for more details of these criteria).

Of all patients initially recruited 14 were excluded because they answered less than 80% in any of the questionnaires. With regard to STAI-T outcomes no patient was excluded because none scored above 3rd quartile; that is, all are below the value 45. Therefore, the study included 81 cancer patients submitted by clinical indication to the ^18^F-FDG PET/CT low dose scan.

### 2.3. Measurements

#### 2.3.1. Scan Experience Questionnaires (SEQ)

With the purpose of evaluating which variables are related to the anxiety of patients we used two Scan Experience Questionnaires (SEQ): Pre and Post Scan (see “Appendix”). The questions of each questionnaire were based on a Portuguese larger survey used in 232 patients who underwent PET/CT [15]. We only used questions relevant to the purpose of this study. The SEQ Pre Scan offers information about the following domains (Table 1): demographic and clinical details about the patient, major patients' concerns, and information offered the day before ^18^F-FDG PET/CT low dose scan.

The SEQ Post Scan affords information in four domains (Table 1): concerns, patient's experience during the procedure, patient's evaluation of the information provided by the professionals prior to the scan, and patient's overall satisfaction about the department.

Patient answered each item of the domain Information on the day before ^18^F-FDG PET/CT low dose scan on SEQ Pre Scan and the domains of Scan Experience, Information on ^18^F-FDG PET/CT low dose day, and Department on SEQ Post Scan in a Likert scale. Higher values indicated a more positive assessment. The questions related to demography, clinical domain (SEQ Pre Scan), and concerns (SEQ Pre and Post Scan) included various possible answers.

*Cronbach's Alpha* coefficients were used in order to evaluate the extent to which the questions of the subscales of the SEQ measure the same concept. The following results were verified: 0.794 to the domain of Information on the day before ^18^F-FDG PET/CT low dose scan, 0.711 to the perception of the scan achievement, 0.643 to the evaluation of the information during the scan, and 0.660 to the perception of overall satisfaction of the department.* Cronbach's Alpha* values obtained showed reasonable internal consistency (0.8 > *α* ≥ 0.7) except for the domains related to the evaluation of the information during the scan and to the perception of overall satisfaction of the department where the values are questionable (0.7 > *α* ≥ 0.6). These results could be due to the low number of questions in these domains [16].

#### 2.3.2. Spielberger State-Trait Anxiety Inventory (STAI)

To assess the anxiety degree we used Spielberger STAI [17], a standardized psychologic evaluation already adapted into Spanish [18] and Portuguese [19].

The STAI is one of the best-established anxiety measures [8, 17, 19], having been used in many studies in several fields of health research. State anxiety (STAI-S) evaluates how the patient feels in that particular situation or moment (e.g., I feel calm; I am angry; I feel very under pressure) and it reflects how threatening a person perceives his environment while in it. The trait anxiety (STAI-T) evaluates how patients “generally feel” (i.e., “I am a steady person”; “I lack self-confidence”) [17]. Participants are asked to rate themselves on each item on the basis of a 4-point Likert scale, ranging from* not at all* (0) to very* much so* (4) for the STAI-S and from* almost never *to* almost always* for the STAI-T. At the end the scores obtained in each test range from 0 to 60, higher values indicate increased anxiety level, while lower scores indicate decreased anxiety level. There is no cut-off value in these tests.

The descriptive statistics was used as a standard measure in order to evaluate STAI-S questionnaires, where the overall assessment is obtained through the sum of items, ranging between 0 and 60 points. The cut-off point for the exclusion of some patients was the third quartile of the scale, that is, the value 45.

### 2.4. Procedure

On the day before the scan, two NM Departments in the Iberian Peninsula gave all patients oral information related to medication, exercise, fasting, and length of time they would need as far as ^18^F-FDG PET/CT low dose was concerned.

On the exam day, all selected cancer patients were contacted by one of researchers who informed patients about the study and gave them an informed consent form to let them know about the study procedures.

The patients filled the SEQ Pre Scan, STAI-S, and STAI-T forms in a separate room, before radiopharmaceutical injection to conduct the ^18^F-FDG PET/CT low dose scan. Later, oral procedural and sensory information was given. Procedural information includes the need to inject a very small amount of radiopharmaceuticals and to rest quietly after the injection and scan procedures: positioning, immobilization, and duration of scan. Sensory information comprises the need to have a cannula into one of the veins in the back of hand or arm, to stay alone in the scanning room, and to lie on his or her back on a narrow bed. Patients were told that the bed moves through the scanner and they would be given a blanket in order to be comfortable.

After collecting data in ^18^F-FDG PET/CT low dose scan, all patients completed the SEQ Post Scan and STAI-S. Only questionnaires in which more than 80% of the questions were answered by the patients were included in this study [19].

### 2.5. Statistical Analyses

Data analyses were carried out using the Statistical Packages for the Social Science, SPSS, version 22.0 for Windows. Descriptive analyses of the study sample were performed. The Shapiro-Wilk test was used with the objective of assessing the normality of data. Results are considered significant at the 5% significance level (*p* < 0.05).

Mann–Whitney* U* test was used to compare the state of anxiety between patients who perform the scan for the first time and those who had already performed previously the scan and between genders. Kruskal-Wallis test was used to compare anxiety among education levels and among the reasons that lead to the examination. Spearman correlation coefficient was used to evaluate the correlation between anxiety (before and after scan) and the various domains of SEQ. Wilcoxon test was used to compare STAIT-S before and after scan. Because normality assumption was not verified (*p* < 0.05) and, moreover, has detected the presence of outliers, nonparametric statistic was used.

## 3. Results

Out of the 81 cancer patients included in the study, 43 (53.1%) were female and 38 (46.9%) were male, with a mean age of 55 ± 14 years (range, 18–79 years). As regards education levels, 17 (20.9%) have compulsory education, 10 (12.3%) secondary education, 23 (28.3%) bachelor's degree, and 31 (38.3%) other levels of education. 77 (95.1%) of the patients knew the reason of the exam. 31 (38.3%) of the patients were made to restage the clinical condition, 22 (27.2%) to initial staging, 20 (24.6%) to assess response to treatment, and 8 (9.8%) to exclude cancer recurrence. 38 (46.9%) carried out the exam for the first time.

According to Table 2, for the majority of patients, ^18^F-FDG PET/CT low dose results are the main cause of anxiety either before or after the scan. Most patients consider that the information provided on the phone on the day before the ^18^F-FDG PET/CT low dose appointment and during the scan in the NM Department was completely understandable (70.5% and 75.3%, resp.) and very useful (70.5% and 72.6%, resp.). About 85.7% of the evaluated patients proved extremely pleased with the service and 89.6% felt treated with dignity and respect.

When one variable moves higher or lower, the other variable moves in the same direction with the same magnitude.

The following significant correlations with low intensity (*p* < 0.05) and positive direction were detected: STAI-S Pre Scan and STAI-S Post Scan (*r*_*S*_ = 0.280, *p* = 0.011); STAI-S Post Scan and the domain of Scan Experience (*r*_*S*_ = 0.266, *p* = 0.022); the domain Information on the day before ^18^F-FDG PET/CT low dose and the domain Information on ^18^F-FDG PET/CT low dose scan day (*r*_*S*_ = 0.373, *p* = 0.006); the domain Information on the day before ^18^F-FDG PET/CT low dose and the domain Overall Satisfaction of Department (*r*_*S*_ = 0.372, *p* = 0.004); the domain Scan Experience and the domain Information on ^18^F-FDG PET/CT low dose scan day (*r*_*S*_ = 0.397, *p* = 0.001); the domain Scan Experience and the domain Overall Satisfaction of Department (*r*_*S*_ = 0.239, *p* = 0.040); the domain Overall Satisfaction of Department and the domain Information on ^18^F-FDG PET/CT low dose scan day (*r*_*S*_ = 0.528, *p* = 0.000). As significant correlations were obtained in the positive direction these results indicate that higher values in one of the variables are related to high values of the other.

Statistically significant differences of anxiety between genders (*p* > 0.05) and between patients who carried out the scan for the first time and those who had already previously performed the scan (*p* > 0.05) were not detected (before and after scan). Statistically significant differences in the state of anxiety among the various levels of education (*p* > 0.05) or among the reasons that led to the scan (*p* > 0.05) were also not detected.

As far as patient age and anxiety are concerned, there were not identifiable significant correlations between ages and STAI-S before (*r*_*S*_ = 0.025, *p* = 0.709) and after (*r*_*S*_ = 0.046, *p* = 0.484) scan.

Table 3 shows the descriptive measures of the STAI-S and reports statistically significant differences in STAI-S before and after scan (*p* < 0.0001), verifying that STAI-S scores at Post Scan are significantly higher than STAI-S scores at Pre Scan.

## 4. Discussion

Anxiety is a common form of distress that people who suffer from an oncology disease are likely to experience. However, there are few published evidences specifically related to the experience of anxiety among patients in imaging studies [20].

The purpose of this study was to research cancer patients' anxiety regarding ^18^F-FDG PET/CT low dose examination and to explore the main reasons that generate anxiety among these patients.

Great levels of anxiety were detected both before and after ^18^F-FDG PET/CT low dose performance (STAI mean > 30). No statistically significant differences were found in the association between the social-demographic variables (i.e., gender, age, and level of education), the reason for the test, and the state anxiety levels before and after the scan. Other studies [15, 20] found great anxiety in male patients who went through ^18^F-FDG PET/CT low dose during the initial staging or during the evaluation of the recurrence of a tumor. The number of patients in our study could account for these differences; however, further researches with larger samples are needed in the future.

The first aim of anxiety management should be to assess the nature and controllability of anxiety faced by patients. In our study, the main reason, for more than two thirds of all patients predisposed to the experience of anxiety, was related to concerns with the result coming from scanning, and, consequently, an uncontrollable matter. Comparable results had been shown by Abreu et al. [15] with oncology outpatients performing ^18^F-FDG PET/CT low dose scan and also by Domènech et al. [3], with patients receiving radioiodine treatment or undergoing a sentinel lymph node in the NM Department. Another study in an imaging department of a cancer center, by Ollivier et al. [21], also found that the most frequent reason for being worried was anxiety about the results.

The patients' concern with ^18^F-FDG PET/CT low dose results may well explain why there were not significant differences in the state of anxiety among patients who went through scanning for the first time and those who had undergone it previously. These findings pointed out that the experience of ^18^F-FDG PET/CT low dose does not minimize its emotional impact in cancer patients. Previous research has showed that repeated experience with Magnetic Resonance Imaging (MRI) simulated decrease in the levels of anxiety in claustrophobic patients' [22]. However, in our study, this does not happen with most patients who had previously been through one scan. Literature suggests [23] that this experience is not enough to differentiate these patients from others who undergo the exam for the first time. Clinical conditions can also account for these results. In fact, cancer patients often experience emotional distress, including anxiety [24] and, particularly, medical imaging can be pointed out by cancer patients as a threat [20].

Contrary to findings from previous studies [15], in our study the levels of anxiety were higher after the ^18^F-FDG PET/CT low dose scan. This increase in the state of anxiety could be related to the anticipation of the results, since patients are aware that the outcome of the examination can determine the severity of the disease (in the case of initial staging or restaging clinical condition), the efficacy of treatment (e.g., chemotherapy and radiotherapy), or the recurrence of cancer. This recognition suggests that the uncertainty of scan results has great influence on the patients' experience of anxiety [23]. Alternatively, these levels could show the feeling of insecurity [23] or exhaustion and discomfort after an intensive period on a rigid body position, especially with the arms positioned over the head, in a restricted space [11, 25, 26]. It remains to be determined whether these anxiety levels increased after patients leave the uptake room or anxiety increases during image acquisition. Because anxiety levels had the potential to cause motion artifacts, the quality of ^18^F-FDG PET/CT low dose and the accuracy of diagnostic decision making can be affected [5, 8]. Future work is required to deepen this topic, for example, by the introduction of physiological measures during the image acquisition of the ^18^F-FDG PET/CT low dose [3].

The information provided, either on the phone on the day before the examination or on the ^18^F-FDG PET/CT low dose day in the NM Department, was classified mostly as totally understandable and as very useful. Most patients rated that the contact made by NM health professionals the day before the scan as being very appropriate (70.5%). That determines the importance of establishing an individualized relationship from the first time [21, 27, 28].

The way information was provided either before or during ^18^F-FDG PET/CT low dose scan is highly associated with satisfaction of patients with NM Department. It has been indicated by other studies in NM Departments [3, 11, 13, 15, 27, 29] that satisfaction is mainly influenced by the impression given by the service organization and by the good performance of professionals. Reyes-Pérez et al. [13] showed that patients have a clear perception of health professionals and of the quality of service, whether they are treated with dignity and respect.

The data from our research also illustrate that although patients are satisfied with NM Department and with the quantity and quality of information that was given by the staff, these aspects are not sufficient to reduce anxiety levels, especially after ^18^F-FDG PET/CT low dose. Similar results were found by Abreu et al. [15] with cancer patients and by Carlson et al. [24] with women awaiting breast biopsy. It seems that sensorial and procedural information that was transmitted to cancer patients was adequate, but uncertain outcomes have more influence on their distress than the procedure itself. Therefore, health professionals also need to focus on nonpharmacological strategies that allow patients' to feel more reassurance during ^18^F-FDG PET/CT low dose imaging [5, 8]. Bradley et al. [5] showed that improving communication between patient and staff, through the use of tangible devise, helps to lower anxiety levels in cancer patients undergoing ^18^F-FDG PET/CT low dose scan. Vogel et al. [8] proved that the use of audiovisual intervention in the ^18^F-FDG PET/CT low dose uptake room allows lowering patient anxiety, and Nightingale et al. [12] reach the same conclusion with an introduction of environmental distractions such as music in cardiac patients. These seem to be effective strategies but require further evidence in larger samples of cancer patients performing ^18^F-FDG PET/CT low dose.

Abreu et al. [15] previously studied anxiety before and after ^18^F-FDG PET/CT scan in oncological patients. However, considering participants and measurements, our study adds some modifications that could explain the differences of results in the two studies. In our sample, most patients who underwent the scan had previous experience in PET/CT scan, therefore already knowing the procedure. In the study done by Abreu et al. [15], 71% of the patients underwent the scan for the first time. The unfamiliarity could explain why the patients feel more anxious before the PET/CT scan. The measures of anxiety used in the two studies were different. While Abreu et al. [15] used a thermometer, with a 10-point Likert-type scale in which subjects were asked to rate their feelings of anxiety, in our study, the anxiety was measured using the STAI, a standardized anxiety questionnaire that has been widely used in medical image procedures [8, 17, 19]. Despite the anxiety thermometer showing positive correlation with the STAI results [30], our findings reinforce the need for further investigation in this area. Considering that, to the best of our knowledge, these are only two studies that measure anxiety before and after a PET/CT scan; the measurement of anxiety levels with thermometer and STAI may prove useful in adding valuable insights of anxiety levels experienced by oncological patients who undergo a PET/CT scan.

This present study has some limitations. Although the questionnaire used to evaluate patients' ^18^F-FDG PET/CT low dose experience had already been used in a previous study, the survey had not been valued with regard to reliability and validity. As far as the Scan Experience Questionnaire Pre Scan is concerned, a few of the questions were answered by less than 81 patients, yet the response rate was never under 80%; these missing data and the small number of patients in total sample could also constrain the validity of this study. Nevertheless, there are few studies concerning oncology patients' experience of anxiety undergoing ^18^F-FDG PET/CT low dose scan and therefore this study can contribute further knowledge to improve patient management at NM Department.

## 5. Conclusions

Cancer patients attending for ^18^F-FDG PET/CT low dose are likely to experience levels of anxiety not only before the scan but also after the scan. Concerns about scan results were pointed out as the main factor for that emotional reaction.

Health professionals at Nuclear Medicine Department need to be aware of the patients' levels of anxiety at all times, even when there are no other signs of distress.

Although patients were satisfied with the information and the care provided by NM's team, findings suggest that, as far as anxiety management is concerned, patients seem to require support of professionals. Nonpharmacological techniques that address uncertainty could be significant for patients and need to be explored by nurses or/and NM's technology professionals.

## Figures and Tables

**Table 1:** Domains of the Scan Questionnaire Experience (SQE) questionnaires: before and after procedure.

Questionnaire	Domains	Question number
Pre Scan	Demographic and clinical	1, 2, 3, 4, 5, 6
	Concerns	7
	Information on the day before ^18^F-FDG PET/CT low dose scan	8.1, 8.2

Post Scan	Concerns	1
	Scan Experience	2, 3
	Information on ^18^F-FDG PET/CT low dose scan day	4.1, 4.2, 5
	Department	6, 7

**Table 2:** Outcomes of SEQ before and after scan.

Variables	Before scan *n* (%)	After scan *n* (%)
Reason for anxiety	67 (82.7%)	61 (75.3%)
Scan procedure	8 (11.9)	4 (6.6)
Results	53 (79.1)	53 (86.9)
Illness	2 (3.0)	1 (1.6)
Other	4 (6.0)	3 (4.9)
Information understanding	61 (75.3)	73 (90.1)
More or less understandable	3 (4.9)	4 (5.5)
Understandable	15 (24.6)	14 (19.2)
Completely understandable	43 (70.5)	55 (75.3)
Utility of the information	61 (75.3)	73 (90.1)
Not very useless	1 (1.6)	0.0
More or less useful	2 (3.3)	2 (2.7)
Useful	15 (24.6)	18 (24.7)
Very useful	43 (70.5)	53 (72.6)
Comfortability to ask questions		77 (95.1)
No, never	—	1 (1.3)
Yes, few times	—	2 (2.6)
Yes, sometimes	—	3 (3.9)
Yes, almost of times	—	17 (22.1)
Yes, always	—	54 (70.1)
Response to questions/doubts		77 (95.1)
Hardly adequate	—	1 (1.3)
More or less adequate	—	8 (10.4)
Adequate	—	28 (36.4)
Very adequate	—	40 (51.9)
Need more information?		77 (95.1)
Yes	—	4 (5.2)
No	—	73 (94.8)
Treatment with dignity and respect		77 (95.1)
Almost	—	8 (10.4)
Always	—	69 (89.6)
Satisfaction with department		77 (95.1)
Not very satisfied	—	1 (1.3)
Satisfied	—	10 (13.0)
Very satisfied	—	66 (85.7)

SEQ: Scan Experience Questionnaire.

**Table 3:** Comparison of STAI-S before and after scan.

STAI-S	Mean	SD	Median	Test statistics^a^	
				*z*	*p*
Before scan	31.099	5.16	31.1	−4.172	0.000
After scan	33.91	4.19	33.0		

STAI-S: Spielberger State-Trait Anxiety Inventory.

^a^Wilcoxon signed-ranks test: *p* < 0.005.

## Appendix

*Scan Experience Questionnaire (SEQ)*

*Pre Scan Experience Questionnaire (SEQ Pre Scan)*

*Information*

1. Gender:
    □ Female
    □ Male
2. Age: —
3. Education:
    □ Compulsory Education
    □ High School
    □ Bachelor's
    □ Master Degree
    □ PhD
    □ Other: —
4. Do you know the name of the scan you are going to have?
    □ Yes
    □ No (If your answer is “no”, please go to question 5)
5. If your answer is “yes”, please state the name below:
6. —
7. Do you know why you are having this scan?
    □ Yes
    □ No (If your answer is “no”, please go to question 6)
  (5.1) If yes, please tick the reason why:
      □ Initial Staging
      □ Characterisation
      □ Assess response to treatment
      □ Exclude recurrence
      □ Other —
8. Is this the first time you are having this scan done?
    □ Yes
    □ No (If your answer is “no” please go to question 8)
9. What concerns you the most?
    □ Scan procedure
    □ Results
    □ Illness
    □ Other —

*Service*

(8) Considered your scan appointment,
  (8.1) How would you rate the information provided to you in terms of understanding?
    Please mark the side of the scale that most reflects your opinion with a cross (X)
  (8.2) How would you rate the information provided to you in terms of helpfulness?
    Please mark the side of the scale that most reflects your opinion with a cross (X)

*Post Scan Experience Questionnaire (SEQ Post Scan)*

1. What are your concerns?
    □ Scan procedure
    □ Results
    □ Illness
    □ Other —

*Scan Experience*

(2) How would you rate the scan procedure?
    Difficult 1 2 3 4 5 Easy
    Negative experience 1 2 3 4 5 Good Experience
(3) How would you describe the scan that you have just had done?
    Uncomfortable 1 2 3 4 5 Comfortable
    Weary 1 2 3 4 5 Tolerable
    Not in control 1 2 3 4 5 In control
    Claustrophobic 1 2 3 4 5 Not claustrophobic

*Information*

(4) How would you rate the information you were provided with in terms of:
  (4.1) *Comprehension*: Please mark the side of the scale that most reflects your opinion with a cross (X)
  (4.2) *Helpfulness*: Please mark the side of the scale that most reflects your opinion with a cross (X)
(5) How would you rate the answers given to you by the staff?
    Please mark the side of the scale that most reflects your opinion with a cross (X)

*Service*

(6) Do you believe the staff treated you with dignity and respect during your time within the Nuclear Medicine service?
    Please mark the side of the scale that most reflects your opinion with a cross (X)
(7) As a whole, how satisfied are you with the PET department?
    Please mark the side of the scale that most reflects your opinion with a cross (X)
